# The cancer-testis Sp17 and ASP are expressed in the ciliated hepatic foregut cysts

**DOI:** 10.1186/2051-1426-1-S1-P52

**Published:** 2013-11-07

**Authors:** Fabio Grizzi, Sonia Di Biccari, Barbara Franceschini, Vladimir Osipov, Everardo Cobos, Maurizio Chiriva-Internati

**Affiliations:** 1 Humanitas Clinical and Research Center, Rozzano, Italy; 2 Department of Anatomical Pathology, Labtests Auckland, New Zealand; 3 Texas Tech University Health Science Center Medical School, Lubbock, TX, USA

## 

Although ciliated hepatic foregut cysts (CHFCs) are retained benign lesions, a case of squamous cell metaplasia and three cases of squamous cell carcinoma arising from CHFCs has been reported. The potential of malignant transformation make it necessary the identification of new biomarkers for more accurate management of CHFCs. Here we investigate the expression of Sperm protein 17 (Sp17) and AKAP-associated sperm protein (ASP) in ciliated cells of two surgically resected CHFCs. CHFCs taken from two patients who went to the Medical College of Wisconsin were investigated. Two-micrometer thick sections were processed for immunohistochemistry with primary anti-Sp17 or -ASP antibodies. To verify whether Sp17 and ASP were co-expressed and co-localized within ciliated cells, further two-micrometers thick sections were processed for dual immunofluorescence and subsequently analyzed with a Leica TCS SP5 confocal microscope. To investigate the specificity of the antibodies, spermatozoa were collected from fertile human volunteers donors. Cell lysates were electrophoresed and transferred onto nitrocellulose membranes. Membranes were incubated with the primary and secondary antibodies and then the bands were visualized with Opti-4CN Substrate kit. β-actin was used as the control protein. CHFCs were found immunopositive for Sp17 and ASP. Both proteins were localized to the cytoplasm of ciliated cells lining the cysts, and in their cilia. Confocal microscopy demonstrated that both Sp17 and ASP overlapped in the same region of the cells. Here we first demonstrate the expression of the cancer-testis antigens Sp17 and ASP in ciliated cells of two CHFCs. Although CHFCs are retained rare benign lesions, one case of squamous cell metaplasia and three cases of squamous cell carcinoma arising from CHFCs have been reported. Sp17 has been detected in oral and esophageal squamous cell carcinomas, and involved in the malignant transformation and metastatic progression of murine squamous cell carcinoma. Further characterization of Sp17 and ASP in CHFCs may provide significant clues for understanding the molecular mechanisms underlying their predisposition to develop squamous cell carcinomas and may reveal new findings on the biology and management of CHFCs.

